# Practical guidance on intensification of insulin therapy with BIAsp 30: a consensus statement

**DOI:** 10.1111/j.1742-1241.2009.02192.x

**Published:** 2009-11

**Authors:** A G Unnikrishnan, J Tibaldi, M Hadley-Brown, A J Krentz, R Ligthelm, T Damci, J Gumprecht, L Gerő, Y Mu, I Raz

**Affiliations:** 1 Department of Endocrinology and Diabetes, Amrita Institute of Medical SciencesKochi, India; 2 Queens Diabetes & Endocrinology, Fresh MeadowsNY, USA; 3 The Surgery, Thetford, Norfolk Primary Care TrustNorfolk, UK; 4 Department of Diabetes, Southampton General Hospital, Southampton University Hospitals NHS TrustSouthampton, UK; 5 EHM Clinic, HoofddorpThe Netherlands; 6 Division of Endocrinology, Diabetes and Metabolism, Istanbul University Cerrahpasa Medical FacultyIstanbul, Turkey; 7 Department and Clinic of Internal Medicine, Diabetology and Nephrology, Silesian University of MedicineKatowice, Poland; 8 First Department of Internal Medicine, Semmelweis UniversityBudapest, Hungary; 9 Department of Endocrinology, PLA General HospitalBeijing, China; 10 Internal Medicine, Hadassah HospitalJerusalem, Israel

## Abstract

**Background:**

Basal insulin and premix insulin are commonly prescribed first-line insulin therapies for patients failing to maintain glycaemic control on oral therapy. When control on these insulins starts to drift, premix analogues, such as biphasic insulin aspart 30/70 (BIAsp 30), are a simple and effective tool for intensification as they can be injected up to three-times daily (TID). However, at present, international recommendations for intensification of insulin therapy using premix analogues are limited and specific guidance on dosing is not available for many scenarios.

**Methods:**

In October 2008, an international expert panel met to review the current guidelines for insulin intensification with BIAsp 30 in patients with type 2 diabetes, with the aim of developing practical guidance for general and specialist practitioners.

**Results:**

Simple treatment algorithms have been developed for (i) patients on basal insulin (human or analogue) once daily or twice daily (BID) who need intensification to BIAsp 30 BID, and (ii) patients on BIAsp 30 once daily or BID who can be intensified to BIAsp 30 BID or TID. As well as these algorithms, specific guidance has been provided on dose transfer (from basal insulin to BIAsp 30), dose split (when intensifying from once daily to BID), and combination oral therapies. In addition, a guide to dose titration is included.

**Conclusions:**

The guidelines presented here should enable general or specialist practitioners to use BIAsp 30 to intensify the insulin therapy of patients failing on basal insulin or BIAsp 30 once or twice daily.

**What's known**

Intensification of failing insulin therapy can be achieved with BIAsp 30, dosed up to three-times-daily. At present, international recommendations for insulin intensification using premix analogues are limited and specific guidance on dosing is not available for many scenarios. In October 2008, an international, independent, expert panel met to review the current guidelines for insulin intensification therapy with BIAsp 30 with the aim of developing international practical guidance for general and specialist practitioners.

**What's new**

Treatment algorithms are presented to help physicians intensify insulin therapy in patients with type 2 diabetes: from basal insulin OD or BID to BIAsp 30 BID, and from BIAsp 30 OD and BID to BIAsp 30 BID and TID, respectively. Randomised controlled trials and observational studies available on PubMed, involving insulin therapy being intensified with BIAsp 30, were reviewed to help provide guidance on injection frequency, dose transfer and titration.

## Introduction

Type 2 diabetes has reached pandemic proportions across the world and the problem continues to grow (1,2). Type 2 diabetes is a progressive disease, characterised by diminishing β-cell function in the context of insulin resistance, driven by obesity (3–5). Impaired glucose tolerance precedes type 2 diabetes and, by the time of clinical diagnosis, patients have lost about half of their β-cell insulin-producing capacity (3,6).

Therapy for type 2 diabetes needs to be steadily intensified in line with the disease progression. Once insulin therapy has been initiated – following the failure of lifestyle changes and oral therapy to keep patients in glycaemic control – there is often a need for intensification from basal insulin or a once-daily (OD) regimen of premix insulin (comprising both basal and prandial insulin components), as these are two commonly prescribed first-line insulin therapies (7).

Although basal insulin, in combination with oral antidiabetics (OADs), is an effective first insulin therapy for patients with poorly controlled type 2 diabetes (8), its efficacy eventually reaches a limit in some patients because, while fasting blood glucose may be at target, postprandial hyperglycaemia may continue to rise and contribute to overall glycaemic levels (9). In the large, international, PRESENT observational study, the average baseline HbA_1c_ in patients receiving basal insulin (analogue or human) was greater than 9.3% (10), possibly at least in part because of impairment of the second phase insulin release resulting from β-cell glucotoxicity as diabetes progresses (4).

Intensified insulin therapy, which includes a rapid-acting prandial component, is therefore appropriate for these patients. Similarly, patients who are failing to maintain glycaemic control on OD analogue premix need intensification to twice daily (BID) to address the postprandial glucose (PPG) excursions after more than one meal per day (11,12). In the 1-2-3 study by Garber et al., 41% of patients with type 2 diabetes who were prescribed the analogue premix, biphasic insulin aspart 30/70 (BIAsp 30, comprising 30% prandial insulin aspart and 70% basal protaminated aspart), achieved HbA_1c_ < 7.0% on an OD regimen over 16 weeks. However, when the BIAsp 30 regimen was intensified to BID and three-times daily (TID) (as necessary), 70% and 77% of patients, respectively, were able to reach this glycaemic goal (12).

International data from routine clinical practice show that glycaemic control in patients with type 2 diabetes is poor on average, even in patients using insulin: almost 50% of patients in the IMPROVE observational study had HbA_1c_≥ 9.0% at the baseline visit (13). Improving treatment and disease management in type 2 diabetes is therefore crucial if long-term vascular complications are to be minimised (14–16), and intensification of failing insulin therapy is a key step in this process.

At present, international recommendations for intensification of insulin therapy using premix analogues are limited. The American Association of Clinical Endocrinologists’ (AACE) guidelines (17) cover the following:

- Transition from a long-acting insulin analogue to a premixed insulin analogue BID.
- Transition from a OD premixed insulin analogue to a BID premixed insulin analogue.

In both scenarios, the recommendations are as follows: (following 1 : 1 dose transfer from basal insulin) divide the total daily dose into two equal doses; give half before breakfast, the other half before dinner; titrate to goal based on self-monitored blood glucose data and diet history; the largest meal will require a larger proportion of insulin; reduce the total dose by 20% if the patient experiences recurrent hypoglycaemia.

The AACE guidelines (17) thus do not cover the possible intensification from BID premix analogue to TID premix analogue. The International Diabetes Federation (IDF) guidelines (18) mention premixes as viable intensification options but offer no specific guidance.

The BIAsp 30^1^ EU label has the indication for progressing from OD to BID and from BID to TID, but again no specific dosing guidelines are given for intensification. A recent consensus statement from the UK recommended premix analogues BID (intensifying to TID as required) as a treatment option for patients with type 2 diabetes switching from basal insulin (19). The initial dose was recommended to be 80% of the final basal dose with titration to target over 14 days. However, these guidelines fail to include guidance on how the dose should be split and titrated (19). New international guidelines that cover all appropriate scenarios for insulin intensification with premixed analogues are therefore needed.

As the diabetes pandemic grows, primary care physicians will need to treat an increasing number of patients with type 2 diabetes because there will be too many cases for specialists to deal with (20). Guidelines for insulin intensification therefore need to be straightforward, comprehensive and easily implemented.

BIAsp 30 is the most prescribed analogue premix and consequently has the largest evidence base in terms of randomised controlled trials (RCTs) and observational data. It follows that BIAsp 30 is therefore the analogue premix most likely to be used for insulin intensification, both from basal insulin and from BIAsp 30 regimens: OD to BID and from BID to TID. In October 2008, an independent international expert panel – comprising the authors o7f this report – met to review the current guidelines for insulin intensification therapy using BIAsp 30 in patients with type 2 diabetes, with the aim of developing international practical guidance for general and specialist practitioners.

## Which patients need intensified therapy? Clinical evidence for intensification with BIAsp 30

Patients who need intensified insulin therapy can essentially be grouped into two categories: those who started insulin with basal therapy and can no longer maintain glycaemic control, and those using BIAsp 30 OD or BID and failing to maintain adequate glycaemic control.

## Patients failing on basal insulin

Initiating insulin therapy with a basal insulin analogue in patients failing on OAD therapy can be effective (21,22), but intensification may be needed long-term. Few studies have addressed the question of what happens to glycaemic control in patients with type 2 diabetes failing to maintain glycaemic goals on basal insulin, after a switch to BIAsp 30. One RCT, the PREFER study, randomised 719 patients previously treated with two OADs with, or without, basal insulin to either BIAsp 30 BID or basal–bolus therapy (insulin detemir and insulin aspart) (23). After 26 weeks of therapy, patients previously treated with basal insulin showed a reduction in HbA_1c_ of 0.75% (baseline level for the BIAsp 30 group was 8.40%). Although previous basal insulin dose was not reported, the total daily BIAsp 30 dose increased by 0.16 U/kg (from 0.47 to 0.63 U/kg) from week 3 to week 26, with a 50/50 breakfast/dinner dose split (23).

Other evidence comes from large observational studies: PRESENT and IMPROVE. These international, non-interventional studies have reported on the effectiveness and safety profile of BIAsp 30 in routine care in patients from a variety of prestudy therapies, including basal insulin. In the PRESENT analysis, glycaemic control at baseline was poor in this patient group, with HbA_1c_ > 9.3% for those previously treated with human or analogue basal insulin (10). After 6 months of BIAsp 30 therapy, HbA_1c_ decreased by a mean of 1.42% and 1.60%, respectively. In terms of dosing, prestudy basal insulin doses were 0.46 for human and 0.34 U/kg for analogue. When the switch to BIAsp 30 was made, doses were transferred, on average, approximately 1 : 1 for those coming from human basal (mean total baseline BIAsp 30 dose: 0.50 U/kg) and 1 : 1.3 for those coming from analogue basal (mean total baseline BIAsp 30 dose: 0.45 U/kg). During the 6-month observation period, doses underwent very little titration: final doses were 0.56 and 0.48 U/kg, respectively (10). The increase in dose when patients transferred from analogue basal insulin to BIAsp 30 did not, however, have corollaries in terms of hypoglycaemia; the rates of major and minor hypoglycaemia were reduced following BIAsp 30 therapy compared with rates on analogue basal insulin (major: 1.1–0.03, p < 0.05; minor: 2.9–2.2 episodes/patient/year, not statistically significant, p>0.05) (10).

In the largest observational study to date of BIAsp 30 in routine care, IMPROVE, patients who were switched from basal insulin to BIAsp 30 were, again, in poor glycaemic control. Mean HbA_1c_ was over 9.0% and patients had been diagnosed with type 2 diabetes, on average, more than 11 years previously (24). After 26 weeks of BIAsp 30 therapy, reductions in HbA_1c_ were −1.64% in patients previously on human basal insulin, and −1.83% in those previously on analogue basal insulin. When switching to BIAsp 30, the transfer of dose was 1 : 1.2 on average (0.33–0.40 U/kg), but patients previously on OD basal insulin were started on a lower BIAsp 30 dose than those previously on BID basal insulin (0.36 and 0.44 U/kg, respectively). The majority of patients (82%) were transferred to a BID BIAsp 30 regimen, regardless of prior basal insulin injection frequency. The dose increase over the observation period was similar in both groups (0.14 vs. 0.13 U/kg) (24).

To summarise, when BIAsp 30 BID was started following basal insulin therapy in routine care, the dose was transferred either 1 : 1 (if human basal) or 1 : 1.3 (if analogue basal), without any safety concerns and resulted in improved glycaemic control. When switching from OD basal insulin, the starting BIAsp 30 dose was smaller than when switching from BID basal insulin, giving an average dose transfer of 1 : 1.2. In addition, data from RCTs have shown that BID BIAsp 30 administration resulted in a 50 : 50 breakfast/dinner dose distribution.

## Patients failing on OD or BID BIAsp 30

Initiating insulin therapy with BIAsp 30 OD is also a successful strategy for improving glycaemic control in patients with type 2 diabetes failing on oral therapy (11,25–27). However, as demonstrated in the 1-2-3 study (12), BIAsp 30 OD will generally only get a minority of patients to the HbA_1c_ target of < 7.0%, but the proportion is increased when dosing is intensified to BID and, if necessary, TID. In this study, 100 patients with type 2 diabetes, previously treated with OADs (with or without basal insulin), were initiated with, or switched basal insulin therapy to, BIAsp 30 OD for 16 weeks. After this time, 21% of patients reached the IDF (18) HbA_1c_ target of < 6.5% and left the study. The remaining patients were intensified to BIAsp 30 BID and, after 16 weeks, to TID if this target had not been reached. This intensification strategy enabled 41%, 70% and 77% of patients on OD, BID and TID to reach HbA_1c_ < 7.0%, respectively (12). The daily insulin dose for patients who achieved the target HbA_1c_ of ≤ 6.5% on BIAsp 30 OD was 0.60 U/kg. For patients who finished the study on BIAsp 30 BID, the total dose almost doubled (due to the extra injection and the relatively aggressive titration algorithm used in this study), with a mean dose split close to 50/50 (0.51/0.64 U/kg breakfast/dinner). For those who finished on BIAsp 30 TID, the dose split was 38/16/46% breakfast/lunch/dinner (0.58/0.25/0.70 U/kg respectively).

Even when the total daily dose with BIAsp 30 TID was smaller (0.59 U/kg), as in the REFORM study (27), the breakfast/lunch/dinner dose split was virtually the same: 34/17/49% (0.20/0.10/0.29 U/kg, respectively). In this study, 101 patients inadequately controlled on OAD combination therapy were randomised to repaglinide 6 mg/day or metformin 2 g/day in combination with BIAsp 30 OD (6 U). If targets were not met [fasting plasma glucose (FPG) 4.0–6.0 mmol/l, HbA_1c_ < 6.5%], patients were intensified to BIAsp 30 BID and then TID at 3, 6 or 9 months. After 12 months, 42% of patients were receiving BIAsp 30 TID. Their mean HbA_1c_ was 7.0%, and 26% of patients achieved HbA_1c_ < 6.5% (27).

The observed doubling of the BIAsp 30 dose when intensifying from OD to BID in the 1-2-3 study (12) was also seen in the trial by Bebakar et al. (11). Here, 191 patients with type 2 diabetes, previously insulin-naïve, were randomised 2 : 1 to BIAsp 30 OD or to an optimised OAD regimen. After 13 weeks, HbA_1c_ was reduced by a significantly greater amount with BIAsp 30 OD than with optimised OADs (−1.16% vs. −0.58%, p<0.001), but only 25% of patients on BIAsp 30 OD reached HbA_1c_ < 7.0%. Those patients with HbA_1c_ > 8.5% or FPG > 7 mmol/l at this point were intensified to BIAsp 30 BID for a further 13 weeks. At the end of the study, HbA_1c_ was reduced by −1.34% in those on BIAsp 30 BID and by −1.24% in those who had remained on BIAsp 30 OD. The starting insulin doses for those who finished on BIAsp 30 OD or BID were very similar: 0.17 and 0.16 U/kg, but patients who intensified to a BID regimen finished the 26-week trial with a BIAsp 30 dose approximately double that of those who remained on an OD regimen: 0.22 vs. 0.43 U/kg (11).

To summarise, in treat-to-target intensification studies, the total dose of BIAsp 30 increased considerably following the consecutive intensification from OD to BID to TID. The dose distribution of BID BIAsp 30 administration was close to 50 : 50, while in studies where patients had intensified to a TID regimen, the highest dose of BIAsp 30 was given at dinner, followed by the doses at breakfast and lunch.

On the basis of an assessment of the published data, combined with many years of clinical experience, the international expert panel agreed on the following guideline for the intensification of insulin therapy using BIAsp 30.

## Practical guidelines for insulin intensification with BIAsp 30

### Switching from basal insulin OD or BID to BIAsp 30 BID

**Figure 1:**
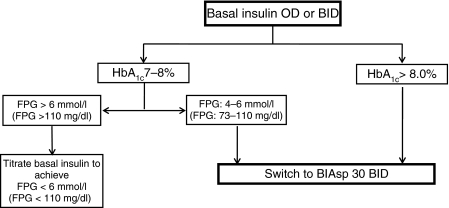
A simple algorithm for the intensification of basal insulin therapy once daily (OD) or twice daily (BID) (analogue or human) to biphasic insulin aspart 30/70 (BIAsp 30) BID. FPG, fasting plasma glucose

A simple algorithm for switching patients from OD or BID basal insulin (analogue or human) to BIAsp 30 BID is shown in Figure 1. Regardless of basal regimen, if a patient has HbA_1c_ higher than 8.0%, they should be transferred to BIAsp 30 BID. If HbA_1c_ is moderately elevated (between 7.0% and 8.0%) but FPG is within the normal range (4–6 mmol/l), the suboptimal overall glycaemia is probably caused by elevated PPG, thus the patient should be transferred to BIAsp 30 BID as it provides prandial coverage as well. If, however, HbA_1c_ is between 7.0% and 8.0%, and FPG is higher than 6 mmol/l, the existing basal insulin dose(s) can be titrated further until the patient achieves FPG below 6 mmol/l. If recurrent hypoglycaemia limits uptitration of the basal dose, or the daily dose reaches 0.5 U/kg (insulin units per kg body weight), switching to BIAsp 30 BID can be considered.

When switching a patient from basal insulin OD or BID to BIAsp 30 BID, the points in Box 1 provide some practical guidance.

**Box 1**

Practical guidance for switching from basal insulin OD or BID to BIAsp 30 BID

1 : 1 Total dose transfer to BIAsp 30
Split the dose 50 : 50 prebreakfast and predinner
Titrate the dose preferably once a week
Discontinue sulfonylureas (SUs)
Continue metformin
Consider discontinuing thiazolidinediones (TZDs) as per local guidelines and practice
Administer BIAsp 30 just before meals

### Intensification with BIAsp 30: from OD to BID and from BID to TID

**Figure 2:**
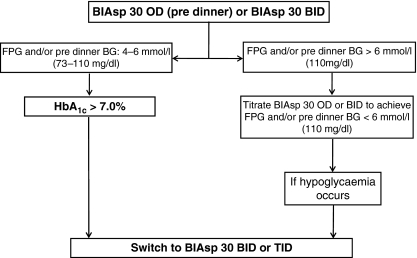
A simple algorithm for intensifying therapy from biphasic insulin aspart 30/70 (BIAsp 30) once (OD) or twice daily (BID) to BIAsp 30 twice or three-times daily (TID). FPG, fasting plasma glucose; BG, blood glucose

An algorithm for intensifying therapy from BIAsp 30 OD or BID to BIAsp 30 BID or TID is shown in Figure 2. If a patient receiving BIAsp 30 OD or BID has FPG (with or without predinner blood glucose measurement) within the normal range (4–6 mmol/l), but has HbA_1c_ higher than 7.0%, the suboptimal overall glycaemia is probably caused by elevated PPG after a meal not covered by BIAsp 30, thus they should be transferred to BIAsp 30 BID or TID (i.e., the addition of just one daily injection). If, however, FPG (with or without predinner blood glucose measurement) is higher than 6 mmol/l, the existing BIAsp 30 dose(s) (OD or BID) should be titrated until the patient achieves FPG below 6 mmol/l. If while doing so hypoglycaemia^2^ occurs, the patient should be intensified to BIAsp 30 BID or TID (i.e., the addition of just one daily injection).

When intensifying a patient’s therapy from BIAsp 30 OD or BID to BIAsp 30 BID or TID, the points in Boxes 2 and 3 provide some practical guidance.

**Box 2**

Practical guidance for switching from BIAsp 30 OD to BID

Split the OD dose into equal breakfast and dinner doses (50 : 50)
Titrate the doses preferably once a week according to the algorithm below
Discontinue SUs
Continue metformin
Consider discontinuing TZDs as per local guidelines and practice
Administer BIAsp 30 just before meals

**Box 3**

Practical guidance for switching from BIAsp 30 BID to TID

Add 2–6 U or 10% of total daily BIAsp 30 dose before lunch
Down-titration of morning dose (−2 to 4 U) may be needed after adding the lunch dose
Titrate the doses preferably once a week according to the algorithm below
Continue metformin
Consider discontinuing TZDs as per local guidelines and practice
Administer BIAsp 30 just before meals

### Titration algorithm for implementing the above guidelines

This algorithm is taken from the INITIATE study (28) and the current NovoMix 30 EU label (available online at: http://www.emea.europa.eu/humandocs/PDFs/EPAR/Novomix/H-308-PI-en.pdf)

**Table:** 

Preprandial blood glucose value		Dose change
< 4.4 mmol/l	< 80 mg/dl	−2 U
4.4–6.1 mmol/l	80–110 mg/dl	0
6.2–7.8 mmol/l	111–140 mg/dl	+2 U
7.9–10.0 mmol/l	141–180 mg/dl	+4 U
> 10.0 mmol/l	> 180 mg/dl	+6 U

When using this titration algorithm to adjust BIAsp 30 doses after intensifying basal insulin therapy to BIAsp 30 BID, or intensifying BIAsp 30 OD or BID to BIAsp 30 BID or TID, the following guidance should be noted:

- The lowest of three previous days’ premeal levels should be used.
- Always change the meal-time dose preceding the measurement.
- The dose should not be increased if hypoglycaemia occurs during these days.
- Dose adjustments can be made once a week until target is reached.
- Only one dose at a time should be changed: the evening dose should be titrated first, followed by the breakfast dose and finally the lunch dose as appropriate.

## Considerations for dosing and titration of BIAsp 30

### When to down-titrate

Down-titrate the dose if major or recurrent minor hypoglycaemia occurs (the United Kingdom Prospective Diabetes Study defined minor hypoglycaemic events as those for which the patient was able to self-treat the symptoms, unaided, while major hypoglycaemic events were those that required third-party help or necessitated medical intervention) (14).

### Patient demographics

- Guidance is aimed at the typical patient with type 2 diabetes.
- These guidelines assume no metabolic decompensation (diabetic ketoacidosis, extreme hyperglycaemia, fluctuating glucose levels).
- These guidelines may not be applicable in special situations like pregnancy, acute coronary events, patients treated in intensive care units, sepsis and any other critical illnesses.

### Other clinical insights

- When transferring a patient from biphasic human insulin to BIAsp 30, start with the same dose and regimen. When dose titration and further intensification are needed, follow the algorithm given above. A recent study shows that patients can safely and effectively self-titrate BIAsp 30 using an algorithm (29).
- Patients with a high body mass index (BMI) are likely to require higher doses of BIAsp 30 than those with a lower BMI and/or the elderly, who may be more insulin-sensitive.
- Multiple doses of insulin are easier to administer using injection pen-type devices (30).
- When the daily insulin dose in a OD regimen nears 40–50 U, intensifying the regimen to BID is a safer way to proceed than simply increasing the dose further, as the dose can be split into two equal doses, which reduces the chance of hypoglycaemia. Each of these doses can then be titrated.
- For patients receiving BIAsp 30 TID, data from RCTs indicate that the dose distribution should approximate the ratio 2 : 1 : 3, breakfast:lunch:dinner (or 33/17/50%).
- BIAsp 30 TID may be a useful alternative to basal–bolus therapy for some patients, as fewer daily injections are required and only one insulin and one device need be used, eliminating the potential for mixing up insulins and hence incorrect dosing.
- Weight gain is a potential barrier to insulin therapy in patients with type 2 diabetes; patients need to have realistic expectations and manage potential weight gain with a regimen of healthy diet and exercise. Continuing metformin therapy might help minimise unwanted weight gain (31).

## Footnotes

1 Novo Nordisk A/S, Bagsværd, Denmark.

2 Hypoglycaemia is defined by the United Kingdom Prospective Diabetes Study as a minor event if the patient is able to self-treat the symptoms, unaided, and a major event if third-party help is required or it necessitates medical intervention (14).
